# Robot-assisted bladder diverticulectomy in adults: a systematic review and pooled analysis of perioperative and functional outcomes

**DOI:** 10.1007/s11701-026-03959-5

**Published:** 2026-09-15

**Authors:** Christos Costa, Foteini Moniati, Christoforos Demosthenous

**Affiliations:** 1 Department of Urology, Churchill Hospital, Oxford University Hospitals NHS Foundation Trust, Oxford, UK; 2 Department of Medicine, University Hospitals Birmingham NHS Trust, Birmingham, UK; 3 Barts and the London School of Medicine and Dentistry, Queen Mary University of London, London, UK

**Keywords:** Bladder diverticulum, Diverticulectomy, Robot-assisted surgery, Bladder outlet obstruction, Minimally invasive surgery

## Abstract

Robot-assisted bladder diverticulectomy (RABD) is used for symptomatic acquired bladder diverticula, but evidence is dispersed across small single-centre series and the only dedicated systematic review dates from 2010. We reviewed contemporary perioperative and functional outcomes of RABD. Following a protocol registered on the Open Science Framework (https://doi.org/10.17605/OSF.IO/54CFT), PubMed, Embase, the Cochrane Library, Scopus and Web of Science were searched from inception to 30 August 2026 (initial search June 2026, re-run and broadened for this version), following PRISMA 2020. Eligible studies were original series of five or more adults undergoing robot-assisted bladder diverticulectomy reporting extractable outcomes. Two reviewers independently screened, extracted data and appraised risk of bias with the Joanna Briggs Institute checklist for case series, with third-reviewer adjudication. Binary outcomes were pooled as proportions with Wilson 95% confidence intervals (CI); continuous outcomes were summarised as patient-number-weighted descriptive values, because mixed median/mean reporting and clinical heterogeneity precluded a formal pooled-effect meta-analysis. Twelve studies (146 patients) were included and all outcomes were extracted from the full-text reports. A transperitoneal route was used throughout. There were no conversions to open surgery (0/129; 95% CI 0–2.9%). Major complications (Clavien–Dindo ≥ III) occurred in 3.0% (4/135; 95% CI 1.2–7.4%). Patient-number-weighted descriptive values (combining study-level medians and means, and therefore not a pooled mean) were: operative time 163 min, blood loss 99 mL, length of stay 3.6 days and catheter duration 7.7 days. Symptom scores and post-void residual improved in every reporting series, significantly in five. The single non-randomised comparison with open surgery reported fewer major complications after RABD (5% [1/20] vs. 50% [3/6], p = 0.007), but the open arm comprised only six patients and this finding should not be regarded as comparative evidence. Across these series, RABD was feasible with low reported short-term morbidity in selected patients: no conversions to open surgery were recorded in the studies reporting conversion status, the major-complication rate was low, and symptom scores and post-void residual improved in every series that measured them. A minority of patients had an incidentally detected intradiverticular tumour; oncological outcomes were not an endpoint of this review. Evidence remains limited by small, heterogeneous, mostly retrospective series, so these findings should be read as descriptive; prospective comparative data are warranted.

**Supplementary Information:**

The online version contains supplementary material available at https://doi.org/10.1007/s11701-026-03959-5.

## Introduction

Bladder diverticula are outpouchings of bladder mucosa through the muscular wall. Most are acquired and arise in older men as a consequence of bladder outlet obstruction (BOO), commonly from benign prostatic enlargement; a minority are congenital. Many are asymptomatic and need no treatment, but surgery is indicated for refractory lower urinary tract symptoms (LUTS), recurrent urinary tract infection, stones, ureteric obstruction, incomplete emptying or an intradiverticular tumour. Malignancy is reported in a meaningful minority of resected diverticula, and intradiverticular tumours pose particular staging difficulty because the muscularis propria is typically absent [1, 2].

Diverticulectomy has been performed by open, endoscopic, laparoscopic and, more recently, robotic approaches. Laparoscopy offered reduced blood loss and shorter hospitalisation compared with open surgery [3, 4], and the wristed instrumentation and three-dimensional vision of the robotic platform are well suited to the reconstructive suturing this operation requires. Since the first robotic reports in 2006–2007 [5, 6], experience has accumulated but remains dispersed across small single-centre series using heterogeneous techniques (extravesical, transvesical and trans-diverticular) and variable strategies for managing the underlying BOO. The only dedicated systematic review is now more than a decade old and was restricted to case reports [7]. We therefore performed an updated systematic review and pooled analysis of the perioperative and functional outcomes of RABD.

## Materials and methods

### Protocol and registration

This review was designed, conducted and reported in accordance with the Preferred Reporting Items for Systematic Reviews and Meta-Analyses (PRISMA) 2020 statement [8]; the completed PRISMA 2020 checklist is provided as Supplementary Table S6. The review protocol — specifying the review question, eligibility criteria, outcomes and synthesis approach — was registered on the Open Science Framework (OSF; https://doi.org/10.17605/OSF.IO/54CFT) during the review, after the initial search and at the screening stage; this timing is stated in the Limitations.

### Search strategy

**Fig. 1:**
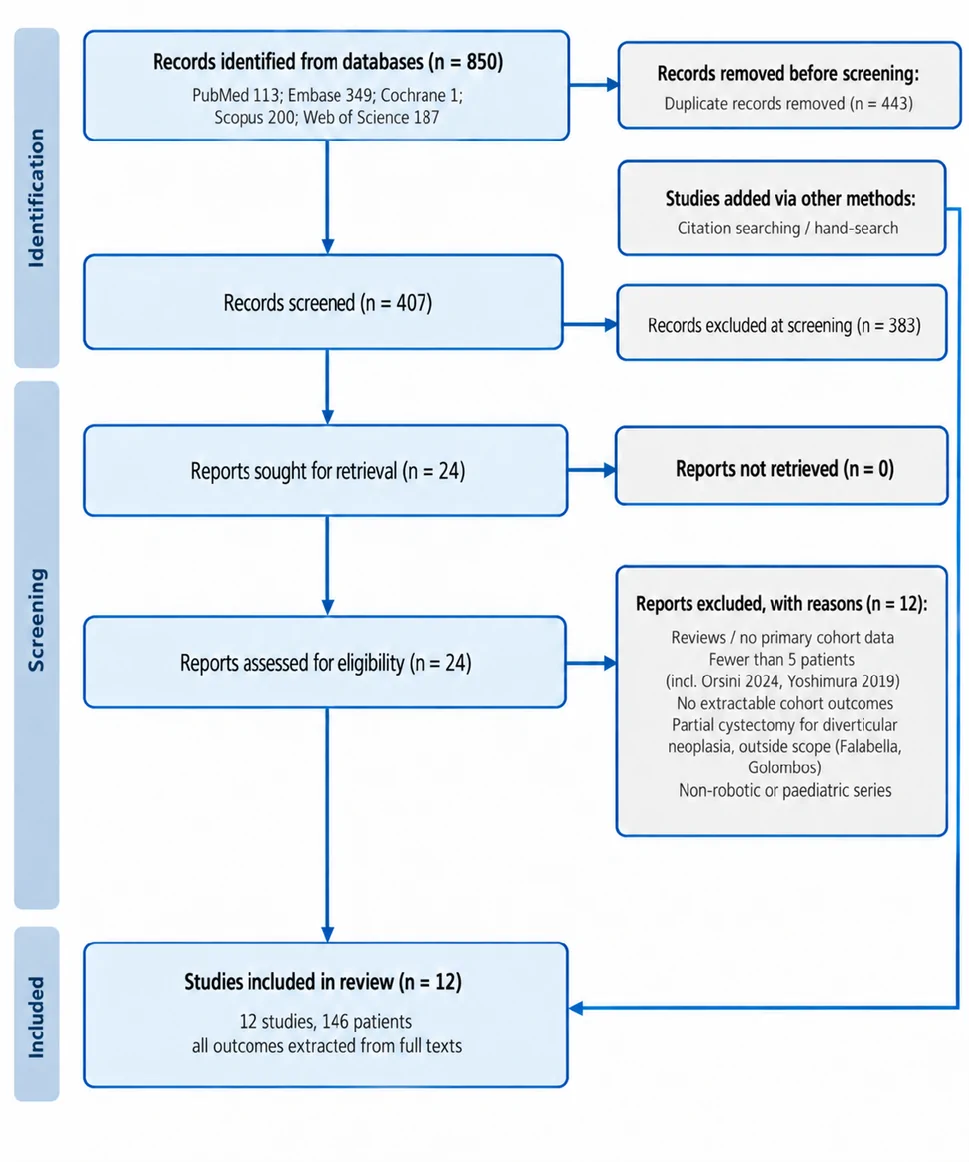
PRISMA 2020 study-selection flow diagram. A total of 850 records were identified from five databases (PubMed 113, Embase 349, Cochrane CENTRAL 1, Scopus 200, Web of Science 187); after removal of 443 duplicates, 407 records were screened, 383 were excluded at title/abstract, and 24 reports were assessed at full text, all of which were retrieved; 12 were excluded with reasons, leaving 12 studies (146 patients). Every assessed report is itemised with its decision in Supplementary Table S3

The literature was first searched in June 2026 in PubMed, Embase and the Cochrane Library. For the present version the search was re-run and broadened: PubMed/MEDLINE, Embase, the Cochrane Library (CENTRAL), Scopus and Web of Science were searched from inception to 30 August 2026, without language or date restriction, combining terms for bladder diverticulum/diverticulectomy with robot-assisted surgery; a separate boundary search for robotic partial cystectomy for diverticular tumour was run to document that literature, and reference lists of included studies and prior reviews were hand-searched. The full reproducible strings are provided in Supplementary S1. The search returned 850 records (PubMed 113, Embase 349, Cochrane CENTRAL 1, Scopus 200, Web of Science 187); after removing 443 duplicates, 407 records were screened and 24 reports were assessed at full text (Fig. 1).

### Eligibility criteria

Eligibility was defined using the PICOS framework. Population: adults (≥ 18 years) undergoing robot-assisted excision of a bladder diverticulum for symptomatic (predominantly benign, outlet-obstruction-related) disease. Studies in which resection of a diverticular malignancy was the primary indication and operation (i.e. robotic partial cystectomy for intradiverticular tumour) were excluded; incidental intradiverticular tumours reported within otherwise benign diverticulectomy cohorts did not by themselves exclude a study. Intervention: robot-assisted bladder diverticulectomy (RABD) by any transperitoneal route (extravesical, transvesical or trans-diverticular), with or without concomitant treatment of bladder outlet obstruction. Comparator: none required; open or laparoscopic comparator arms were recorded where reported. Outcomes: primary — conversion to open surgery and major complications (Clavien–Dindo grade ≥ III); secondary — operative time, estimated blood loss, length of stay, catheter duration, symptom score, and post-void residual volume. Study design: original observational studies (prospective or retrospective cohorts and case series) reporting extractable outcomes on ≥ 5 patients. Studies were eligible if they reported at least one pre-specified outcome; each outcome was pooled only across the studies reporting it, so denominators differ between outcomes and are stated throughout. Reviews without primary data, paediatric series, non-robotic series, single case reports, technical or video reports without extractable cohort outcomes, and series of robotic partial cystectomy for diverticular malignancy were excluded. Every identified report is listed with its inclusion or exclusion decision in Supplementary Table S3.

### Study selection and data extraction

Two reviewers (CC, FM) independently screened records and extracted data manually using a standardised form; disagreements were resolved by discussion, with adjudication by a third reviewer (CD). In accordance with the registered protocol, screening, data extraction and risk-of-bias appraisal were performed manually by the named reviewers, without automation or AI-assisted extraction. Extracted variables included design, country, sample size, approach, indication, concomitant procedures, operative time, estimated blood loss (EBL), conversion to open surgery, length of stay (LOS), catheter duration, complications (Clavien–Dindo classification [9]), functional measures (symptom score, post-void residual (PVR)). A single rule was applied to malignancy: where a study reported outcomes separately for a malignant subgroup, only the non-malignant patients were extracted (Gibson [10], from which the 18 non-malignant patients were taken); where a predominantly benign series contained a small number of incidental intradiverticular tumours whose outcomes were not separable at patient level, the cohort was extracted in full and the tumours recorded, because excluding them was not arithmetically possible from the published data. Oncological outcomes were not analysed in either case. Where a value was reported as a median or a mean, the central value and its type were recorded; no value was imputed. All outcomes were extracted from the full-text reports.

### Risk of bias

Two reviewers independently appraised each included study with the Joanna Briggs Institute (JBI) Critical Appraisal Checklist for Case Series [11]; the agreed assessment is presented in Supplementary Table S4. Studies reporting limited methodological detail were scored ‘unclear’ on the affected domains.

### Synthesis

Binary outcomes (conversion to open surgery; major complications, defined as Clavien–Dindo grade ≥ III) were pooled as aggregate patient-level proportions across the studies reporting them, with 95% confidence intervals by the Wilson method. We chose an aggregate proportion in preference to a random-effects pooled proportion because the events were very few (four major complications in total, and none for conversion), several studies had zero events, and with event counts of this size a random-effects model is unstable and its between-study variance cannot be estimated reliably; a single-arm random-effects meta-analysis would convey false precision. This approach does not account for clustering by study, so the confidence intervals are narrower than a model allowing for between-study variation would give, and the pooled proportions should be read as descriptive summaries rather than as estimates of a population parameter. Study-level numerators and denominators for every outcome are reported in Supplementary Table S5 so that readers can inspect the contribution of each study. Continuous outcomes were summarised as patient-number-weighted descriptive values with the range of study-level central values. Because studies mixed medians and means and were clinically heterogeneous, a formal random-effects pooled-effect meta-analysis of continuous outcomes was not undertaken; the weighted figures are descriptive. All twelve included studies were fully extracted from their primary reports, so no secondary-source sensitivity analysis was required. Wilson score intervals were calculated using the standard closed-form expression (z = 1.96) implemented in Python 3.11; because the interval has an exact algebraic form, the values are reproducible in any statistical package.

## Results

### Study selection and characteristics

Twelve series published between 2007 and 2024 were included (Fig. 1; Table 1) [5, 12], 13– [10, 20, 21], contributing 146 patients; all outcomes were extracted from the full-text reports. For one multi-surgeon series that combined benign and malignant indications [10], only the 18 non-malignant patients were extracted, in keeping with the review scope. Most cohorts were predominantly or exclusively male, with a median age in the seventh decade. A transperitoneal route was used throughout, with extravesical, transvesical or trans-diverticular dissection of the diverticular neck. Three predominantly benign series each included two patients with an incidental intradiverticular tumour whose outcomes were not separable at patient level [14, 16, 18], so six of the 146 pooled patients (4.1%) had a tumour-bearing diverticulum; consistent with the review scope these were recorded but not analysed as an oncological endpoint. Where a malignant subgroup was separately reported it was excluded [10], and one report confined to robotic partial cystectomy for diverticular neoplasia was excluded in full as outside scope (Supplementary Table S3).

**Table 1:** Characteristics of included studies

Study	Year	Country	Design	*n*	Approach/indication
Altunrende [13]	2011	USA	Retrospective	6	Extra-/transvesical; benign (2 incidental tumours)
Myer [5]	2007	USA	Retrospective	5	Transperitoneal; benign (BOO)
de Castro Abreu [14]	2014	USA	Retrospective	10	Trans-/extravesical; benign
Davidiuk [15]	2015	USA	Retrospective	16	External/internal; benign (2 incidental tumours)
Tufek [16]	2016	Turkey	Retrospective	9	Extravesical; benign (BOO)
Cacciamani [12]	2018	Italy	Retrospective	6	Extravesical; benign (posterior BD)
Liu [17]	2021	USA	Prospective database	20	Extra-/trans-/trans-diverticular; benign (2 incidental tumours)
Giannarini [18]	2022	Italy	Prospective	16	Extravesical; benign (BOO/BPE)
Develtere [10]	2022	Belgium	Retrospective	23	Transvesical; benign (mostly BOO)
Janardanan [31]	2023	UK	Retrospective	6	Extravesical; benign (refractory LUTS)
Kajaia [32]	2022	Germany	Retrospective	11	Extra-/transvesical; benign (BOO)
Gibson [19]	2024	Australia	Retrospective, multi-surgeon	18	Extravesical; benign subgroup (18 of 28; malignant cases excluded)

*BD* bladder diverticulum, *BOO* bladder outlet obstruction, *BPE* benign prostatic enlargement

### Risk of bias

No study was judged to be at high risk of bias on any JBI domain, but methodological reporting was frequently incomplete and no domain was rated ‘no’. Demographic reporting (Q6) and description of site and setting (Q9) were adequate in every study. The recurring weaknesses were the reporting of inclusion criteria (Q1) and of valid case identification (Q3), each unclear in 11 of 12 studies, and consecutive inclusion (Q4), unclear in 9; complete inclusion of eligible patients (Q5) was unclear in 5. Only one prospective series satisfied all ten domains [19], whereas the multi-surgeon retrospective series was unclear on eight [10]. This pattern — retrospective case series that do not state whether consecutive or complete cohorts were reported — is the principal threat to validity here, since selective inclusion of successful cases would bias the safety and functional estimates favourably. The full appraisal is given in Supplementary Table S4.

### Perioperative outcomes and safety

Pooled outcomes are shown in Table 2. Conversion to open surgery was reported in studies totalling 129 patients, with none observed (0/129; 95% CI 0–2.9%); Janardanan and Kajaia did not report conversion status and do not contribute to this denominator. Major complications (Clavien–Dindo ≥ III) occurred in 3.0% (4/135; 95% CI 1.2–7.4%): a grade IIIb small-bowel obstruction after concomitant simple prostatectomy [15], a grade IIIa cystoscopic clot evacuation [18], a grade IVa pulmonary embolus [10], and a migrated ureteral stent requiring ureteroscopic retrieval in the first reported series (graded Clavien–Dindo IIIb by the present authors, as the original report predates routine Clavien–Dindo grading) [5]; Kajaia [21] reported that complications were not gradable by the Clavien–Dindo system and did not state conversions, so it contributes to the descriptive continuous outcomes but to neither primary-outcome denominator; it met eligibility on the secondary outcomes it did report, and its exclusion from both primary denominators is the reason those denominators (129 and 135) fall short of 146. Patient-number-weighted descriptive values were: operative time 163 min (range 105–232; 146 patients), EBL 99 mL (range 20–250; 130 patients, Cacciamani and Kajaia not reporting), LOS 3.6 days (range 1.6–7.6; 146 patients) and catheter duration 7.7 days (range 2–12.3; 129 patients — Cacciamani and Janardanan did not report it, and Myer was excluded owing to an internal inconsistency in the source between the tabulated and in-text values). Each weighted figure is therefore based only on the studies reporting that outcome, and the contributing denominators are given in Table 2. Concomitant management of BOO — staged or simultaneous transurethral resection or vaporisation of the prostate, or simple/radical prostatectomy — was frequent, reflecting the obstructive aetiology of most acquired diverticula [13, 17–19].

**Table 2:** Pooled peri-operative and safety outcomes (12 studies, 146 patients)

Outcome	Pooled estimate (12 studies, 146 patients)
Conversion to open	0/129 (0%; 95% CI 0–2.9%)
Major complication (Clavien–Dindo ≥ III)	4/135 (3.0%; 95% CI 1.2–7.4%)
Operative time (min)	163 (range 105–232); *n* = 146
Estimated blood loss (mL)	99 (range 20–250); *n* = 130
Length of stay (days)	3.6 (range 1.6–7.6); *n* = 146
Catheter duration (days)	7.7 (range 2–12.3); *n* = 129

Continuous outcomes are patient-number-weighted descriptive values combining study-level medians and means; they are not pooled means and are descriptive only. The range is of study-level central values, and n is the number of patients in the studies reporting that outcome. All studies were extracted from their full-text reports. *CI* confidence interval

### Functional outcomes

Symptom scores and PVR improved in every series that measured them and reached statistical significance in five (Table 3): the International Prostate Symptom Score (IPSS) fell by 57% in one series [15], from 25 to 5 with PVR 195 to 30 mL in another [19], IPSS 19 to 6 with PVR 425 to 49 mL in the largest mixed series [18], IPSS 27.8 to 8 with PVR 249 to 33 mL in a UK series confined to refractory LUTS [20], and a significant post-void residual reduction (742 to 41 mL, *p* = 0.0005) in the non-malignant subgroup of the multi-surgeon series [10]. In a further series the American Urological Association symptom score (18 to 7) and PVR (458 to 214 mL) improved without reaching significance, attributed to the small sample [16]; additional series reported resolution of LUTS and normalisation of PVR at 6 months [13, 17]. Because most cohorts combined diverticulectomy with treatment of the underlying outlet obstruction, this functional improvement cannot be attributed to diverticulectomy alone.

**Table 3:** Functional outcomes by study (pre- to post-operative symptom score and post-void residual)

Study	Symptom score (pre → post)	PVR, mL (pre → post)	Significant
Giannarini [18]	IPSS 25 → 5	195 → 30	Yes (*p* < 0.001)
Liu [17]	IPSS 19 → 6	425 → 49	Yes
Janardanan [31]	IPSS 27.8 → 8	249 → 33	Yes (*p* ≤ 0.001)
Gibson [19]	IPSS pre 20.4 (post NR)	742 → 41	Yes, for PVR (*p* = 0.0005)
Develtere [10]	—	↓120 (post 42)	Not tested
Kajaia [32]	IPSS post 5.6	183 → 22	Not tested
de Castro Abreu [14]	IPSS ↓57%	NR	Yes
Davidiuk [15]	AUA-SS 18 → 7	458 → 214	No (small sample)
Cacciamani [12]	LUTS resolved	Normalised (6 mo)	NR
Tufek [16]	LUTS resolved	Normalised (6 mo)	NR

*IPSS* International Prostate Symptom Score; *AUA-SS* American Urological Association symptom score; *PVR* post-void residual; *NR* not reported. Most cohorts underwent concurrent treatment of bladder outlet obstruction

## Discussion

### Principal findings

Across twelve contemporary series (146 patients, all with data extracted from the full-text reports), robot-assisted bladder diverticulectomy (RABD) was completed without conversion to open surgery in every series that reported conversion status (0/129), with a low major-complication rate (4/135; 3.0%), modest blood loss and short hospitalisation. Symptom scores and post-void residual volume improved in every series that measured them. Because bladder diverticulectomy is an uncommon operation performed across a range of indications [22, 23], individual series are inevitably small; aggregating them provides a descriptive estimate of contemporary RABD outcomes, and the resulting profile is consistent with contemporary reviews of bladder-diverticulum management [7, 22, 24]. A recent scoping review that pooled robotic cases alongside transurethral, laparoscopic and open cohorts reported a comparably low major-complication rate and consistent functional improvement across approaches [24]; the present review is confined to the robotic approach and, to our knowledge, is among the larger robotic-specific syntheses, but is not the largest synthesis of bladder-diverticulum surgery overall. These findings update and extend the only previous dedicated systematic review of the robotic approach, which was limited to case reports [7]; the 2013 narrative review of this operation [25] and the recent scoping review of all surgical approaches [24] are distinct in design and scope.

### Comparison with the literature

The present estimates are concordant with earlier robotic experience: a 2013 review of 44 patients reported a mean operative time of 186 min, blood loss 86 mL and length of stay 2.4 days [25], and single-centre cohorts published since describe similarly low reported morbidity [19, 21]. A further small European cohort combining diverticulectomy with transurethral prostate resection reported comparable outcomes but fell below our five-patient threshold and was not pooled [26]. They also fit the wider trajectory of minimally invasive diverticulectomy. Laparoscopy was shown two decades ago to reduce blood loss, analgesic requirement and hospital stay relative to open surgery [4], and minimally invasive techniques have progressively supplanted open repair [27]; the robotic platform adds the wristed instrumentation and magnified three-dimensional view that reconstruction of the bladder wall in the deep male pelvis demands [3]. The largest single series, a 23-patient transvesical cohort, reported notably short catheterisation (2 days), suggesting that a transvesical route may shorten catheter time relative to extravesical dissection [12]. The only direct comparison with open surgery comes from a single institution, where RABD (*n* = 20) showed lower blood loss (100 vs. 283 mL), shorter catheterisation (12.3 vs. 44 days) and fewer major complications (Clavien ≥ III 5% [1/20] vs. 50% [3/6], *p* = 0.007) than a contemporary open cohort at the same institution [18]; the open comparator arm comprised only six patients (with two grade IIIa events and one peri-operative death), so the percentages rest on very small numbers. These are non-randomised, single-centre data; no randomised trial exists, and no study has compared robotic with conventional laparoscopic diverticulectomy, so the incremental value of the robot over laparoscopy remains unquantified.

### Technical and clinical considerations

Several technical themes recur. Intraoperative identification of the diverticular neck, flattened under pneumoperitoneum, is the pivotal step, and reported adjuncts include retrograde bladder distension, an intradiverticular balloon catheter, flexible cystoscopic transillumination and near-infrared fluorescence [5, 17, 28]. The choice between an extravesical and a transvesical route is largely dictated by diverticulum location: posterior and posterolateral diverticula are well suited to direct extravesical dissection [13, 19], whereas a transvesical route affords controlled access to peri-trigonal or peri-ureteric necks and, in the largest series, was associated with notably short catheterisation [12]. Protection of the ureteric orifice — by preoperative stenting or careful intraoperative identification — is emphasised for peri-ureteric diverticula, and ureteric reimplantation is occasionally required [5, 18]. Single-port robotic platforms are now being applied to this operation, with early transvesical and transabdominal descriptions suggesting feasibility, and represent the most likely direction of future technical refinement [29, 30].

Two clinical principles also emerge. First, because most acquired diverticula are driven by bladder outlet obstruction, durable functional success depends on relieving the obstruction, whether staged or performed concurrently with diverticulectomy [13, 17, 19, 26]; the low complication rate observed even when prostatic surgery was combined is compatible with a single-setting strategy in appropriately selected patients, although these uncontrolled data cannot establish that a combined procedure is as safe as a staged one. Second, a bladder diverticulum may occasionally harbour a tumour — intradiverticular tumours account for approximately 1% of bladder cancers and are difficult to stage because the diverticulum lacks a muscularis propria [1, 2, 31]. We deliberately confined this review to diverticulectomy for symptomatic, predominantly benign disease and did not evaluate oncological outcomes, because the robotic oncological experience with diverticular tumours is largely reported under the term robotic partial cystectomy, which lies outside the inclusion scope of the pooled review. That literature is substantial: a pooled analysis of 498 intradiverticular bladder tumours has summarised their presentation, staging pitfalls and management [31], and robotic excision of tumour-bearing diverticula is described in dedicated series — for example, a single-surgeon cohort of 29 robot-assisted partial cystectomies that specifically analysed the intradiverticular-neoplasm subgroup, with margin status, nodal yield and five-year survival [32]. Where an intradiverticular tumour was incidentally present in the diverticulectomy series included here, standard oncological principles (attention to margins and pelvic lymph-node dissection where indicated) were applied [10, 18], but the numbers are too few and follow-up too short to support any oncological conclusion, and a dedicated review of oncological outcomes would need to search the partial-cystectomy literature.

### Strengths and limitations

Strengths of this review include protocol registration on OSF, a search of five databases with a documented decision for every screened report, extraction of all twelve included studies in full from their primary reports — performed manually and in duplicate, with no study taken from an abstract or a secondary source — duplicate independent risk-of-bias appraisal, and confidence intervals for the clinically critical safety outcomes. The limitations are nonetheless substantial and define how the findings should be read. The included studies are small, heterogeneous and almost all retrospective and single-centre, with a probable bias towards publication of favourable experience; formal statistical assessment of reporting bias (funnel-plot asymmetry or small-study tests) was not appropriate with this number of small single-arm series, so reporting bias was assessed narratively; continuous outcomes mixed medians and means, which precludes a valid pooled-effect meta-analysis, so the weighted figures are descriptive only; the binary outcomes are single-arm proportions without a comparator; for one multi-surgeon series only the non-malignant subgroup could be extracted, and where series combined diverticulectomy with treatment of bladder outlet obstruction the functional gains cannot be attributed to diverticulectomy alone; and follow-up was short and unevenly reported: three series did not state it at all, and among the nine that did it ranged from one month to 18 months (median 6 months). Because durability of functional benefit and diverticular recurrence cannot be judged over such intervals, the functional findings should be read as early post-operative results rather than as evidence of sustained benefit, and no conclusion about recurrence is possible. Registration on OSF (12 July 2026) followed the initial search rather than preceding it. The literature was searched across five databases with a final pre-submission search on 30 August 2026; oncological experience with intradiverticular tumours reported under the term robotic partial cystectomy was deliberately outside scope and is not captured here. The review was confined to diverticulectomy for predominantly benign disease; a minority of included patients nonetheless had an incidental intradiverticular tumour that could not be separated at patient level from the reported cohort outcomes, so the perioperative figures are not exclusively benign, although oncological outcomes were not analysed. Taken together, the overall certainty of evidence is low, and the results should be read as supporting feasibility with low reported short-term morbidity rather than superiority over open or laparoscopic surgery. A prospective, registered, comparative evaluation — ideally multicentre, with standardised outcome reporting, longer follow-up and, where possible, individual-patient-data synthesis — is the necessary next step to define the role of RABD.

## Conclusion

In this synthesis, robot-assisted bladder diverticulectomy was feasible with low reported short-term morbidity in selected patients, with no conversions to open surgery among the studies reporting conversion status, a low rate of major complications, modest blood loss, short hospital stay, and improvement in symptom scores and post-void residual in every series that measured them. The current evidence remains limited by small, heterogeneous, mostly retrospective studies and should be regarded as descriptive; prospective comparative data with longer follow-up are needed to define the role of RABD relative to laparoscopic and open surgery.

## Supplementary Information

Below is the link to the electronic supplementary material.

Supplementary Material 1

## Data Availability

All data analysed in this review are contained within the article and its Supplementary Information; all values derive from the cited published studies. The full data-extraction workbook is available from the corresponding author on reasonable request.

## Declarations

### Use of AI and AI-assisted technologies

AI-assisted tools were used solely for language and copy editing of author-generated text, including improvements to grammar and readability. Study screening, data extraction and risk-of-bias appraisal were performed manually by the named reviewers, in accordance with the registered protocol, without automation or AI-assisted extraction. The authors reviewed and edited all content and take full responsibility for the publication.Review Article (systematic review and pooled analysis).
