# Supplementary material for: Robot-assisted bladder diverticulectomy in adults: a systematic review and pooled analysis of perioperative and functional outcomes
Source: J Robot Surg. 2026 Sep 15;20(1):994. doi: 10.1007/s11701-026-03959-5 (PMC13578062; doi:10.1007/s11701-026-03959-5)
Supplement: Supplementary file 1 [file 11701_2026_3959_MOESM1_ESM.docx]

**Supplementary Material**

*At submission, the following items should be uploaded as separate Online Resources (e.g. ESM_1.pdf) and cited in the text accordingly; they are reproduced here for review convenience.*

**S1. Reproducible search strategies**

*All databases searched from inception to 30 August 2026, with no language or date restriction and no species/human filter applied (screening for adults and humans was performed manually). Main search: 850 records (PubMed 113, Embase 349, Cochrane CENTRAL 1, Scopus 200, Web of Science 187). A separate boundary search was run to document the robotic partial-cystectomy-for-diverticular-tumour literature, which was screened and excluded as outside scope.*

**A. Main search**

**PubMed / MEDLINE (NCBI; 30 August 2026; n = 113)**

```
((("Diverticulum"[Mesh] AND "Urinary Bladder"[Mesh])
  OR ((bladder[tiab] OR vesical[tiab] OR intravesical[tiab])
      AND (diverticul*[tiab] OR diverticulectom*[tiab]))))
AND (robot*[tiab] OR "da Vinci"[tiab]
     OR "Robotic Surgical Procedures"[Mesh] OR "Robotics"[Mesh])
```

**Embase (Embase.com; 30 August 2026; n = 349)**

```
('bladder diverticulum'/exp
  OR ((bladder:ti,ab OR vesical:ti,ab OR intravesical:ti,ab)
      AND (diverticul*:ti,ab OR diverticulectom*:ti,ab)))
AND ('robot assisted surgery'/exp OR 'robotics'/exp
     OR robot*:ti,ab OR 'da vinci':ti,ab)
```

**Cochrane Library / CENTRAL (Wiley; 30 August 2026; n = 1)**

```
((bladder OR vesical OR intravesical)
 AND (diverticul* OR diverticulectom*))
AND (robot* OR "da Vinci"):ti,ab,kw
```

**Scopus (Elsevier; 30 August 2026; n = 200)**

```
TITLE-ABS-KEY(((bladder OR vesical OR intravesical)
  AND (diverticul* OR diverticulectom*))
  AND (robot* OR "da Vinci"))
```

**Web of Science (Clarivate; 30 August 2026; n = 187)**

```
TS=(((bladder OR vesical OR intravesical)
  AND (diverticul* OR diverticulectom*))
  AND (robot* OR "da Vinci"))
```

**B. Boundary search (robotic partial cystectomy for diverticular tumour — screened, excluded as outside scope)**

**Embase n = 39; Cochrane n = 1; Scopus n = 22; Web of Science n = 95**

```
TITLE-ABS-KEY((partial AND cystectom*)
 AND (intradiverticular OR ((bladder OR vesical) AND diverticul*
      AND (tumo* OR cancer* OR carcinoma* OR neoplas*
           OR malignan* OR urothelial)))
 AND (robot* OR "da Vinci"))
[translated to each database's syntax as above]
```

**S2. Note on data handling**

Where an outcome was not reported, the field was left blank rather than imputed. All twelve included studies were extracted from their full-text reports; no study was extracted from an abstract or a secondary source. For one multi-surgeon series [19], only the 18 non-malignant patients were extracted, in keeping with the review scope.

**Supplementary Table S3. Study-selection record with decisions**

*Every robotic bladder diverticulectomy report identified, with decision and reason. All included studies were extracted from their full-text reports.*

| **Report** | **n** | **Decision** | **Reason / basis** |
| --- | --- | --- | --- |
| Altunrende 2011 | 6 | Included | Adult RABD outcome series; full report |
| Myer 2007 | 5 | Included | First adult RABD outcome series; full report |
| de Castro Abreu 2014 | 10 | Included | Adult RABD outcome series; full report |
| Davidiuk 2015 | 16 | Included | Adult RABD outcome series; full report; consecutive |
| Tufek 2016 | 9 | Included | Adult RABD outcome series; full report |
| Cacciamani 2018 | 6 | Included | Adult RABD outcome series; full report; consecutive |
| Liu 2021 | 20 | Included | Adult RABD outcome series; full report; open comparator |
| Giannarini 2022 | 16 | Included | Adult RABD prospective cohort; full report |
| Janardanan 2023 | 6 | Included | Adult RABD outcome series; full report (benign, refractory LUTS); identified on updated search |
| Gibson 2024 | 28 (18 extracted) | Included | Adult RABD outcome series |
| Falabella 2025 | 10 | Excluded | Robotic partial cystectomy for diverticular neoplasia; primary malignant indication, outside scope (cf. Tareen 2008) |
| Develtere 2022 | 23 | Included | Adult RABD outcome series; full-text article (transvesical); 23 patients |
| Eyraud 2013 | – | Excluded | Review without original cohort data |
| Moreno Sierra 2010 | – | Excluded | Systematic review of case reports; no new primary data |
| Macejko 2008 | 2 | Excluded | < 5 patients; technical report |
| Tareen 2008 | 4 | Excluded | Partial cystectomy + diverticulectomy; not BD-specific; < 5 |
| Yoshimura 2019 | 3 | Excluded | < 5 patients |
| Moore 2012 | 5 | Excluded | Identification-technique report; outcomes not separately extractable |
| Ashton 2019 | – | Excluded | Point-of-technique report; no extractable cohort outcomes |
| Thuroff 2012 | – | Excluded | Surgical atlas; no cohort outcomes |
| Hattori 2024 | – | Excluded | Diverticulectomy during radical prostatectomy; technique-focused |
| Kajaia 2022 | 11 | Included | Adult RABD outcome series; full-text article (German); 11 patients |
| Orsini 2024 | 4 | Excluded | Fewer than 5 patients (n = 4); excluded on sample-size criterion |
| Title/abstract exclusions (n = 383) | – | Excluded | Non-bladder diverticula (urethral, oesophageal, calyceal, Meckel, urachal); paediatric series; single case reports; reviews and editorials; robotic procedures other than bladder diverticulectomy |

**Supplementary Table S4. Risk of bias (JBI Critical Appraisal Checklist for Case Series)**

*Q1 inclusion criteria; Q2 condition measured reliably; Q3 valid case identification; Q4 consecutive inclusion; Q5 complete inclusion; Q6 demographics; Q7 clinical information; Q8 outcomes/follow-up; Q9 site/setting; Q10 statistical analysis. Y = yes, U = unclear, N = no.*

| **Study** | **Q1** | **Q2** | **Q3** | **Q4** | **Q5** | **Q6** | **Q7** | **Q8** | **Q9** | **Q10** | **Basis for key ‘unclear’ judgements** |
| --- | --- | --- | --- | --- | --- | --- | --- | --- | --- | --- | --- |
| Altunrende 2011 | U | Y | U | U | U | Y | Y | U | Y | Y | Retrospective chart review; eligibility, consecutiveness and follow-up not specified |
| Myer 2007 | U | Y | U | U | Y | Y | Y | Y | Y | Y | All patients included; consecutiveness/eligibility not explicit |
| de Castro Abreu 2014 | U | Y | U | U | U | Y | Y | Y | Y | Y | Consecutiveness not stated; follow-up reported |
| Davidiuk 2015 | U | Y | U | Y | Y | Y | Y | Y | Y | Y | Explicitly consecutive; pre/post analysis |
| Tufek 2016 | U | Y | U | U | U | Y | Y | U | Y | Y | Small series; follow-up not quantified |
| Cacciamani 2018 | U | Y | U | Y | Y | Y | Y | Y | Y | Y | Consecutive; follow-up and functional outcomes reported |
| Liu 2021 | U | Y | U | U | U | Y | Y | Y | Y | Y | Prospective database; comparator group; consecutiveness not explicit |
| Giannarini 2022 | Y | Y | Y | Y | Y | Y | Y | Y | Y | Y | Prospective, consecutive, explicit eligibility — clearest methodology |
| Janardanan 2023 | U | Y | U | U | Y | Y | Y | Y | Y | Y | Retrospective single-centre; explicit exclusions and 6-month follow-up; consecutiveness/eligibility not fully specified |
| Develtere 2022 | U | Y | U | U | Y | Y | Y | Y | Y | Y | Retrospective single-centre; Clavien-graded outcomes and 9-month follow-up; consecutiveness not stated |
| Kajaia 2022 | U | Y | U | U | Y | Y | U | Y | Y | U | Retrospective single-centre; complications not graded by Clavien–Dindo; functional data partly reported |
| Gibson 2024 | U | U | U | U | U | Y | U | U | Y | U | Full case series; several domains unclear from the available report |

**Supplementary Table S5. Full data extraction**

*Values are medians unless marked m = mean; NR = not reported; n/a = not applicable. † catheter time misprinted in source. ‡ Myer catheter time inconsistent (table 25 d vs text ~14 d).*

| **Study** | **n** | **Approach** | **Indication** | **Age** | **OP (min)** | **EBL (mL)** | **Conv** | **LOS (d)** | **Cath (d)** | **Major ≥III** | **Incidental tumour (n; margin status if stated)** | **FU (mo)** |
| --- | --- | --- | --- | --- | --- | --- | --- | --- | --- | --- | --- | --- |
| Altunrende | 6 | Extra-/transves. | Benign (2 tum) | 61.5 | 232 | 100 | 0 | 3 | 7 | 0 | NR (2 tum) | NR |
| Myer | 5 | Transperitoneal | Benign | 71 | 178 | NR | 0 | 3 | ~14‡ | 1§ | n/a | NR |
| de Castro Abreu | 10 | Trans-/extraves. | Benign | 65 | 210 | 75 | 0 | 2 | 8 | 1 | n/a | 18 |
| Davidiuk | 16 | Ext/int diss. | Benign (2 tum) | 68 | 184 | 50 | 0 | 2 | 11 | 0 | NR (2 tum) | 4.8 |
| Tufek | 9 | Extravesical | Benign | 62m | 186m | 71m | 0 | 5m | 8m | 0 | n/a | NR |
| Cacciamani | 6 | Extravesical | Benign | 66.5 | 112.5 | 25.8 | 0 | 7 | NR† | 0 | n/a | 9 |
| Liu | 20 | Ext/trans/t-div | Benign (2 tum) | 66 | 184m | 100m | 0 | 2.1m | 12.3m | 1 | 0/2 pos | 9 |
| Giannarini | 16 | Extravesical | Benign | 68 | 126 | 20 | 0 | 6 | 5 | 0 | n/a | 6 |
| Janardanan | 6 | Extravesical | Benign | 63.8 | 166 | 75 | NR | 1.6m | NR | 0 | n/a | 6 |
| Kajaia | 11 | Extra-/transvesical | Benign | 64.8 | 212 | NR | NR | 7.6 | 6.5 | NR | n/a | 1 |
| Gibson | 18 | Extravesical | Benign | 61.7 | 105 | 75 | 0 | 2.67 | 10.11 | 1 | n/a | 1.5 |
| Develtere | 23 | Transvesical | Benign | 66 | 140 | 250 | 0 | 3 | 2 | 0 | n/a | 9 |

*§ Myer: migrated ureteral stent requiring ureteroscopic retrieval, graded Clavien–Dindo IIIb.*

**Supplementary Table S6. PRISMA 2020 checklist**

*Item numbers follow the PRISMA 2020 statement (Page et al., BMJ 2021;372:n71).*

| **Item** | **Checklist item** | **Location in this manuscript** |
| --- | --- | --- |
| 1 | Title | Identified as a systematic review in the title. |
| 2 | Abstract | Structured abstract; see PRISMA 2020 for Abstracts items (background, eligibility, sources and date, appraisal tool, synthesis, results, limitations, registration). |
| 3 | Rationale | Introduction: evidence dispersed across small series; only dedicated review dates from 2010. |
| 4 | Objectives | Introduction, final paragraph; PICOS in Eligibility criteria. |
| 5 | Eligibility criteria | Methods — Eligibility criteria (PICOS); benign scope stated; Supplementary Table S3. |
| 6 | Information sources | Methods — Search strategy: PubMed, Embase, Cochrane CENTRAL, Scopus, Web of Science; inception to 30 August 2026; reference lists hand-searched. |
| 7 | Search strategy | Supplementary S1 (full reproducible strings for all five databases, plus boundary search). |
| 8 | Selection process | Methods — Study selection: two independent reviewers, third-reviewer adjudication. |
| 9 | Data collection process | Methods — Study selection and data extraction; standardised form; all data from full texts. |
| 10 | Data items | Methods — Study selection and data extraction (list of extracted variables); Supplementary Table S5. |
| 11 | Risk of bias | Methods — Risk of bias: JBI Critical Appraisal Checklist for Case Series; Supplementary Table S4. |
| 12 | Effect measures | Methods — Synthesis: proportions with Wilson 95% CI; patient-number-weighted descriptive values. |
| 13 | Synthesis methods | Methods — Synthesis; rationale for not performing random-effects meta-analysis; Python 3.11. |
| 14 | Reporting bias | Methods/Discussion — Strengths and limitations: probable publication bias toward favourable experience; formal small-study testing not feasible with fewer than 10 comparable studies. |
| 15 | Certainty assessment | Discussion — Strengths and limitations: overall certainty rated low. |
| 16 | Study selection results | Results — Study selection and characteristics; Fig. 1; Supplementary Table S3. |
| 17 | Study characteristics | Table 1; Supplementary Table S5. |
| 18 | Risk of bias in studies | Supplementary Table S4. |
| 19 | Results of individual studies | Supplementary Table S5; Table 3. |
| 20 | Results of syntheses | Table 2; Results — Perioperative outcomes and Functional outcomes. |
| 21 | Reporting biases | Discussion — Strengths and limitations. |
| 22 | Certainty of evidence | Discussion — Strengths and limitations (low certainty). |
| 23 | Discussion | Discussion — Principal findings, Comparison with the literature, Strengths and limitations, Conclusion. |
| 24 | Registration and protocol | Methods — Protocol and registration: OSF https://doi.org/10.17605/OSF.IO/54CFT (registered 12 July 2026); revised scope and eligibility criteria described in Methods and Discussion. |
| 25 | Support | Statements and Declarations — Funding: none received. |
| 26 | Competing interests | Statements and Declarations — Competing interests: none. |
| 27 | Availability of data | Statements and Declarations — Data availability. |
